# Examining electrometer performance checks with direct‐current generator in a clinic: Assessment of generated charges and implementation of electrometer checks

**DOI:** 10.1002/acm2.13312

**Published:** 2021-06-03

**Authors:** Naoki Kinoshita, Hiroshi Oguchi, Morihito Shimizu, Eiji Kidoya, Hiroki Shioura, Hirohiko Kimura

**Affiliations:** ^1^ Radiological Center University of Fukui Hospital Fukui Japan; ^2^ Department of Radiological and Medical Laboratory Sciences Nagoya University Graduate School of Medicine Nagoya Japan; ^3^ National Metrology Institute of Japan AIST Tsukuba Japan; ^4^ Department of Radiology University of Fukui Hospital Fukui Japan

**Keywords:** clinical electrometer check, electrometer equipped with direct‐current generator, nonlinearity, repeatability

## Abstract

**Purpose:**

Medical physicists use a suitable detector connected to an electrometer to measure radiotherapy beams. Each detector and electrometer has a lifetime (due to physical deterioration of detector components and electrical characteristic deterioration in electronic electrometer components), long‐term stability [according to IEC 60731:2011, ≤0.5% (reference‐class dosimeter)], and calibration frequency [according to Muir et al. (J Appl Clin Med Phys. 2017; 18:182‐190), generally 2 years]; thus, physicists should check the electrometer and detector separately. However, to the best of our knowledge, only one study (Blad et al., *Phys Med Biol*. 1998; 43:2385–2391) has reported checking the electrometer independently from the detector. The present study conducts performance checks on electrometers separately from the detector in clinical settings, using an electrometer equipped with a direct current (DC) generator (EMF 521R) capable of injecting DC (effective range: ±20 pA to ±20 nA) into itself or another electrometer.

**Methods:**

First, to check the nonlinearity of the generated currents from ±20 pA to ±20 nA, charges generated from the DC generator were measured with the EMF 521R electrometer. Next, six reference‐class electrometers classified according to IEC 60731:2011 were checked for repeatability at a current of ±20 pA or a minimum effective indicated value meeting IEC 60731:2011, as well as for nonlinearity within the current range from ±20 pA to ±20 nA.

**Results:**

The nonlinearities for the measured currents were less than ±0.05%. The repeatability for the six electrometers was < 0.1%. While the nonlinearity of one electrometer reached up to 0.22% at a current of –20 pA, all six electrometers displayed nonlinearities of less than ±0.1% at currents of ±100 pA or higher.

**Conclusions:**

This work suggests that it is possible to check the nonlinearity and repeatability of clinical electrometers with DCs above the ±30 pA level using a DC generator in a clinic.

## 1 INTRODUCTION

Radiotherapy dosimeters, specified in IEC 60731:2011,^1^ are measurement systems usually consisting of an ionization chamber, extension cable, and electrometer. According to an addendum to AAPM TG‐51,^2^ the uncertainty in the raw reading measured with these systems, excluding any variability in beam delivery, is due to the electrometer as well as the ionization chamber. Therefore, it is important to develop methods to test clinical electrometers.

All measurement systems have a limited lifetime; therefore, it is necessary to make routine checks of systems used in clinical settings.^2^, ^3^ The addendum to AAPM TG‐51 showed the measurement of polarity correction for a simple QA check of the measurement systems.^2^ This addendum also described a method to monitor the long‐term stability of the systems with a check source or a linac‐beam.^2^ These checks are meant for monitoring the entire system.

Various techniques, such as stereotactic radiosurgery, stereotactic body radiotherapy, and intensity‐modulated radiotherapy, have been introduced in many modern radiotherapy clinics. Consequently, clinical physicists often select the most suitable detector for the measurement object (e.g., small‐field‐, reference‐, relative‐dosimetry, and pre‐treatment dose verification) with various radiotherapy beams; namely, an electrometer is connected to various detectors in these clinical measurements. Each detector and electrometer has a lifetime (due to physical deterioration of the detector’s components and electrical characteristic deterioration in the electrometer’s electronic parts), long‐term stability [according to IEC 60731:2011, ≤0.5% (reference‐class dosimeter)], and calibration frequency (according to recent surveys on reference dosimetry practices,^4^ generally 2 years). Therefore, to ensure accurate measurements, each component of the measurement systems should be checked individually.

While an electrometer plays an important role in clinical dosimetry, to the best of our knowledge, performance checks on an electrometer separated from the detector have been poorly documented.^5^ Therefore, this work examined the feasibility of conducting performance checks on electrometers with a direct‐current (DC) generator in radiotherapy clinics. In this work, charges generated from the DC generator in a clinical setting were first verified. Then, electrometer performance checks were implemented using the DC generator to determine whether it can be used by physicists for electrometer performance checks.

## 2 MATERIALS AND METHODS

Section 2.A summarizes the characteristics of the DC generator used in this work. Section 2.B describes the verification of charges generated from the DC generator. Section 2.C describes the implementation of electrometer checks.

### 2.A Characteristics of DC generator

#### 2.A.1 Range of generated DCs and injection time

**Fig. 1:**
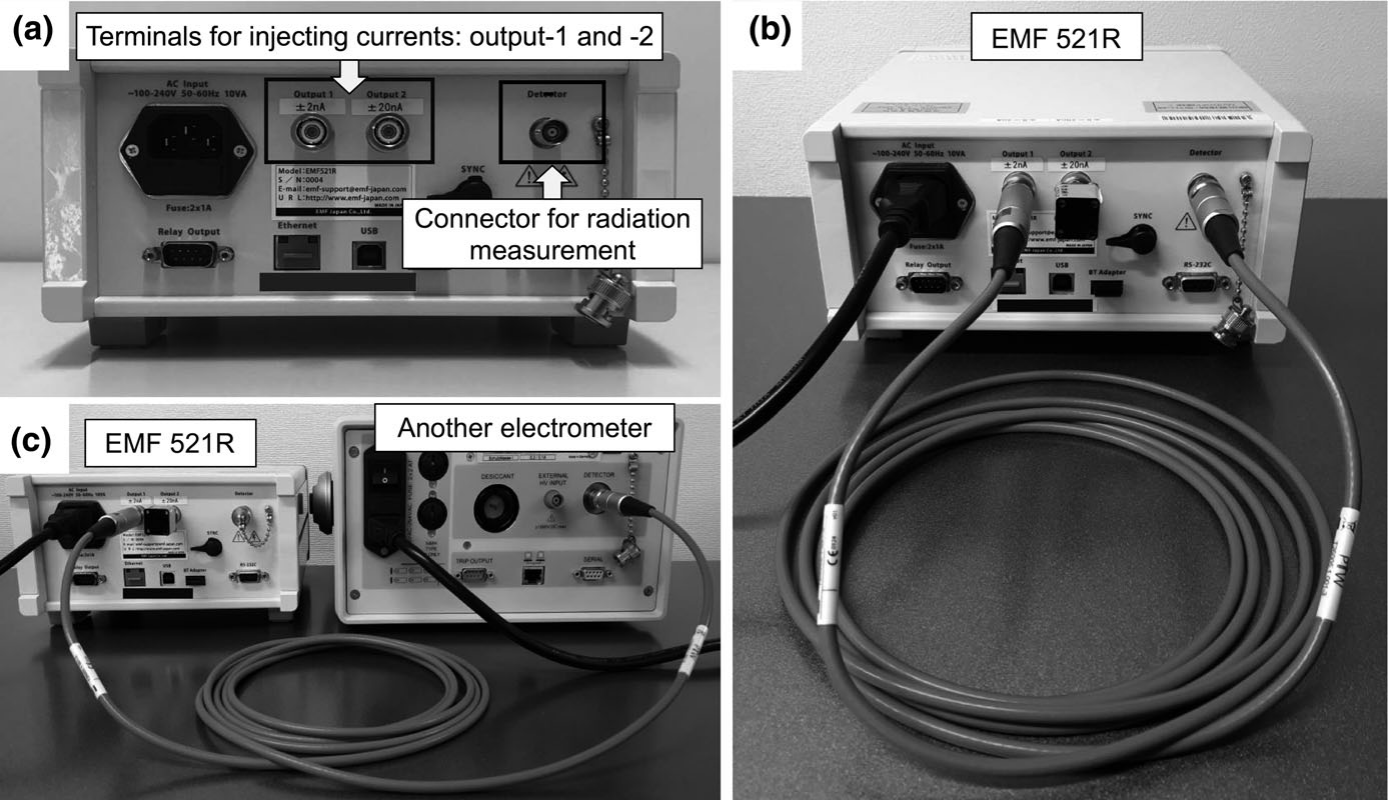
Image of EMF 521R used in this work. This electrometer can transfer constant currents to itself [panel (b)] or to another electrometer [panel (c)] through output‐1 and −2 [panel (a)] for electrometer performance checks.

The DC generator used in this work was equipped with an electrometer [EMF 521R (EMF Japan Co., Ltd., Hyogo, Japan)] that can inject DC into itself or another electrometer for performance checks (Fig. 1). This DC generator had two terminals for injecting DCs: output‐1 and −2 [Fig. 1(a)]. Effective currents from the DC generator ranged from ±20 pA to ±2 nA for output‐1 and from ±200 pA to ±20 nA for output‐2. The injection time of the DC generator can be set in the range of 0.1–1000 s. Because charge is the product of current and time, the DC generator can be utilized as a charge generator as well.

#### 2.A.2 Traceability of the DCs to primary standards

The generated constant currents can be traced to primary standards as follows:

1. The charge (C) and frequency (1/s) composing the currents (A = C/s) were calibrated with in‐house (EMF Japan) electrometers and a universal counter, respectively, at EMF Japan.
2. The in‐house electrometers and universal counter were calibrated using a standard electrometer and frequency standard, respectively, at accredited calibration laboratories that satisfy ISO/IEC 17025 requirements.
3. The voltage source and standard capacitor for the standard electrometer were traceable to the primary standards for DC voltage and capacitance in Japan, respectively. The frequency standard was traceable to the primary standard for frequency in Japan.

#### 2.A.3 Uncertainty in the DCs

The accuracies (*k* = 1) of the DCs from the DC generator were 0.15% and 0.10% for output‐1 and output‐2, respectively. The uncertainty (*k* = 1) in the injection time of the DCs was 0.003%. Table 1 summarizes the uncertainties in charges generated from the DC generator, which were estimated following the Guide to the Expression of Uncertainty in Measurements.^6^ The principal sources of these uncertainties were as follows:

1. In‐house standard device (i.e., electrometer and a universal counter) calibrations: These were obtained from their calibration certificates.
2. Current calibration with the in‐house electrometer: This component corresponded to the uncertainty in the in‐house electrometer reading under the calibration condition [room temperature of 23 ± 1°C and relative humidity (RH) in the range of 20–60%].
3. The DCs at a radiotherapy facility: This source was an uncertainty in the DCs, which were generated at a room temperature of 23 ± 7°C and RH range of 20–60%.

**Table 1:** Uncertainty budget (*k* = 1) for charges generated from the DC generator.

Source	Output‐1 (%)	Output‐2 (%)
1. In‐house standard device calibrations	0.08	0.08
2. Current calibration with in‐house electrometer	0.03	0.03
3. Direct current at a radiotherapy facility	0.12	0.05
Combined uncertainty (*k* = 1)	0.15	0.10

### 2.B Verification of charges generated from the DC generator

To verify the charges generated from the DC generator in realistic clinical situations, the generator was placed in a clinical environment [room temperature of approximately (24 ± 0.5) °C and RH of approximately (32.5 ± 2.5)%].

The charges generated from the DC generator were measured with an EMF 521R electrometer (serial number: X004) [as illustrated in Fig. 1(b)], which was calibrated separately from an ionization chamber in the Association for Nuclear Technology in Medicine [ANTM, which is a JCSS (Japan Calibration Service System) Accredited Calibration Laboratory]. To check the generated‐current nonlinearity, the following currents were measured for 50 s:

1. 20, 200, 1000 [reference point (RP)], and 2000 pA from output‐1,
2. 0.2, 2, 10 (RP), and 20 nA from output‐2.

The raw electrometer readings were multiplied by the electrometer calibration coefficient (*P*_elec_) supplied by ANTM, and the products were assumed to be the measured charges.

The nonlinearities checked here were expressed as percentage deviations [σ (%)] from linearity for the ratio of the measured charges to set charges between the RP and the other points, which were obtained by the following equation:

(1) $$\sigma \left(\%\right)\;=\;100\cdot \left\{\left(\frac{m\cdot Q}{M\cdot q}\right)‐1\right\},$$

where *M* (i.e., measured charge) is produced by *Q* (i.e., set charge at RP) and *m* (i.e., measured charges) is derived from *q* (i.e., set charges at each measurement point except for the RP). These observed nonlinearities would consist of two elements: generated current and measurement instrument nonlinearities, which were within ±0.1% each, according to the specifications. Assuming each of the two uncertainties (each of their probability distributions was assumed to be a rectangular distribution within ±0.1%) were ±0.06% (*k* = 1), the relative combined standard uncertainty in the observed nonlinearities would be 0.09%. Then, whether the observed nonlinearities were within 0.09% was verified. If the observed nonlinearities were 0.09% or less, it was assumed that the generated charge nonlinearities met the performance specified in the specification.

In addition to the nonlinearity checks, differences between the measured charges and set charges were assessed. The uncertainty in the measured charges, which consisted of the raw electrometer reading, *P*_elec_, and the charges generated at the radiation facility, was estimated in accordance with the Guide to the Expression of Uncertainty in Measurements.^6^

### 2.C Electrometer check with the DC generator

Six electrometers, which met the requirements for a reference‐class electrometer described in IEC 60731:2011, were checked. The characteristics of the six electrometers are listed in Table 2.

**Table 2:** Characteristics of the six electrometers checked in this work.

Electrometer	1	2	3	4	5	6
NF in op‐amp	Capacitor	Resistor	Resistor	Capacitor	Resistor	Resistor
Range	L; H	Single	L; H	Single	L; H	L; M; H
ER	L: ±1.0 pA to ±10 nA H: ±0.4 nA to ±2 µA	±4 pA to ±20 nA	L: ±0.4 pA to ±1 nA* H: ±0.4 nA to ±500 nA*	±2 pC to ±10 mC*	L: ±0.4 pA to ±500 pA* H: ±0.4 nA to ±500 nA*	L: ±0.4 pA to ±250 pA* M: ±20 pA to ±25 nA* H: ±2 nA to ±2.5 μA*
EIV	L: ±1.0 pA to ±10 nA H: ±0.4 nA to ±2 µA	±4 pA to ±20 nA	L: ±0.4 pA to ±1 nA* H: ±0.4 nA to ±500 nA*	±2 pC to ±10 mC*	L: ±0.4 pA to ±500 pA* H: ±0.4 nA to ±500 nA*	L: ±0.4 pA to ±250 pA* M: ±20 pA to ±25 A* H: ±2 nA to ±2.5 μA*

Abbreviations: NF in op‐amp, negative feedback in operational amplifier; Capacitor, auto‐discharge integrating capacitor; Range, measuring range; L, Low; M, middle; H, high; ER, effective range of readings; and EIV, effective range of indicated values. Effective ranges of readings and indicated values for the six electrometers corresponded to those described in IEC 60731:2011, with four exceptions (electrometers 3–6). For these, the performances (e.g., repeatability and nonlinearity) were guaranteed in the user’s manuals to be within values marked with *.

In this work, the electrometer performance checks were carried out for repeatability and nonlinearity, which were generally performed according to IEC 60731:2011.^1^ The DCs were injected into the electrometers via the following procedure:

1. According to the instruction for EMF 521R, half of the maximum effective constant currents generated from the DC generator after 1 h of being switched on agreed with those generated after 15 min and 6 h of being switched on, within a range of ±0.02%. Therefore, the DC generator and electrometers were switched on at least approximately 1 h before these tests were started.
2. The electrometers were connected to the DC generator via a low‐noise triaxial cable [Fig. 1(b) and (c)]. The systems (electrometer, cable, and DC generator) were adjusted to compensate for leakage currents by canceling their electrometer readings offset.
3. The DC generator injected DCs into the electrometers. Then, the currents with smaller uncertainties were selected. Output‐2 generated currents of 200 pA and higher, whereas output‐1 generated currents of less than 200 pA.

The electrometer checks were performed under the following conditions: room temperature and RH were in the ranges of 22–27 °C and approximately 40–70%, respectively; changes in the ambient temperature and RH were approximately within ±1 °C and up to 20% (usually about 10%), respectively.

#### 2.C.1 Repeatability

For the repeatability check, constant currents were repeatedly (10 times) injected into the six electrometers for 50 s. The amount of current injected in each case was 20 pA (because the minimum effective output current generated from the generator was ±20 pA) or the minimum effective indicated value^1^ meeting the IEC 60731:2011 standard, if this was larger than ±20 pA. Similarly, while checking the electrometer 4 shown in Table 2, the same process was performed because it was difficult to inject the minimum detectable charge (i.e., 2 pC) into the electrometer.

To evaluate the repeatability for the electrometers, the relative standard deviation of 10 successive readings, expressed as a percentage of the mean indicated value, was calculated.

#### 2.C.2 Nonlinearity

The ranges of current values for nonlinearity checks performed here are listed in Table 3. According to IEC 60731:2011, to assess nonlinearity for an electrometer, the currents injected into it should range as follows: from the minimum effective reading to the maximum effective reading for a single‐range electrometer; from the minimum effective reading on the most sensitive dose‐rate range to the maximum effective reading on the least sensitive dose‐rate range for a multiple‐range electrometer. It was impossible to carry out nonlinearity checks for the ranges described in IEC 60731:2011 because effective currents from the DC generator were in the range of 20 pA to 20 nA. Hence, this study checked nonlinearity from ±20 pA to ±20 nA (or the maximum effective reading, when that value was less than ±20 nA) for all six electrometers.

**Table 3:** Ranges of DC currents for nonlinearity checks performed in this work.

Electrometer	Range	Range of input currents (nA)	RP (nA)
1	Low	±0.02 to ±10	±5
2	Single	±0.02 to ±20	±10
3	Low	±0.02 to ±1	±0.5
4	Single	±0.02 to ±20	±20
5	Low	±0.02 to ±0.5	±0.25
6	Low	±0.02 to ±0.25	±0.125
	Middle	±0.02 to ±20	±10

Abbreviation: RP, reference point.

Different currents in the range of ±20 pA to ±20 nA (or the maximum effective reading, less than ±20 nA), which were spaced at approximately the same interval of the current range expressed as a logarithmic scale, were transferred for 50 s to the six electrometers. Half of the maximum effective reading was taken as the RP for each electrometer (with the exception of electrometer 4, for which ±20 nA was taken as the RP).

These nonlinearities were expressed as percentage deviations from linearity for the ratios of the electrometer readings to set charges between the RP and the other points, which were evaluated using the equation specified in Section 6.3.3 in IEC 60731:2011.

## 3 RESULTS

### 3.A Verification of charges from the DC generator

**Fig. 2:**
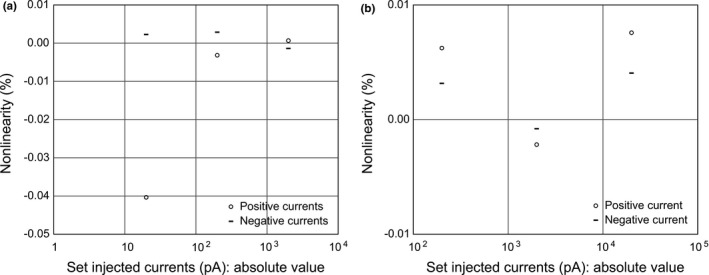
Nonlinearities for measured current from the DC generator: (a) from output‐1 and (b) from output‐2. The y‐axis shows the nonlinearity (%); the x‐axis indicates the injected currents on a logarithmic scale.

Figure 2 shows nonlinearities for measured currents from the DC generator. In all situations investigated here, the nonlinearities were below ±0.05%; the nonlinearities for the measured currents from output‐1 were –0.04% (at 20 pA) or less (well below ±0.01% at each current of above 20 pA), whereas those from output‐2 were well below ±0.01%.

The discrepancies between the measured charges and set charges were within ±0.03%. The uncertainties in these measured charges (*k* = 1) were 0.18% for output‐1 and 0.14% for output‐2 (Table 4).

**Table 4:** Uncertainty budget (*k* = 1) for measured charges.

Source and type of uncertainty	Output 1 (%)	Output 2 (%)
1. Charges generated from the DC generator	0.15	0.10
2. *P*_elec_ for EMF 521R (SN: X004)	0.08	0.08
3. Electrometer reading (EMF 521R SN: X004)	0.05	0.05
Combined uncertainty (*k* = 1)	0.18	0.14

Source 1 is quoted from Table 1. Source 2 was obtained from its calibration certificate issued by the ANTM. Source 3 was derived from the electrometer performances of the EMF 521R, which are listed in IEC 60731:2011.

### 3.B Electrometer check with the DC generator

#### 3.B.1 Repeatability

The repeatabilities for all six electrometers were <0.1%; in fact, all repeatability performances, except for electrometers 3 and 6 (both high range), were 0.05% or less (Table 5).

**Table 5:** Repeatability for the six electrometers.

Electrometer	Range	Injected current (pA)	Mean reading (pA)	Standard deviation (%)
1	Low	−20	−19.98	0.04
		+20	+20.00	0.05
	High	−400	−399.8	0.05
		+400	+399.6	0.05
2	Single	−20	−20.00	0.01
		+20	+20.01	0.01
3	Low	−20	−20.00	0.01
		+20	+20.04	0.02
	High	−400	−401.6	0.07
		+400	+399.7	0.09
4	Single	−20	−19.97	0.03
		+20	+19.96	0.02
5	Low	−20	−20.03	0.05
		+20	+20.03	0.05
	High	−400	−400.4	0.02
		+400	+401	0.02
6	Low	−20	−19.98	0.005
		+20	+20.01	0.05
	Middle	−20	−20.04	0.04
		+20	+19.99	0.03
	High	−2000	−2000.5	0.05
		+2000	+2000.4	0.06

Mean readings and standard deviations were calculated for constant currents injected 10 times successively.

#### 3.B.2 Nonlinearity

**Fig. 3:**
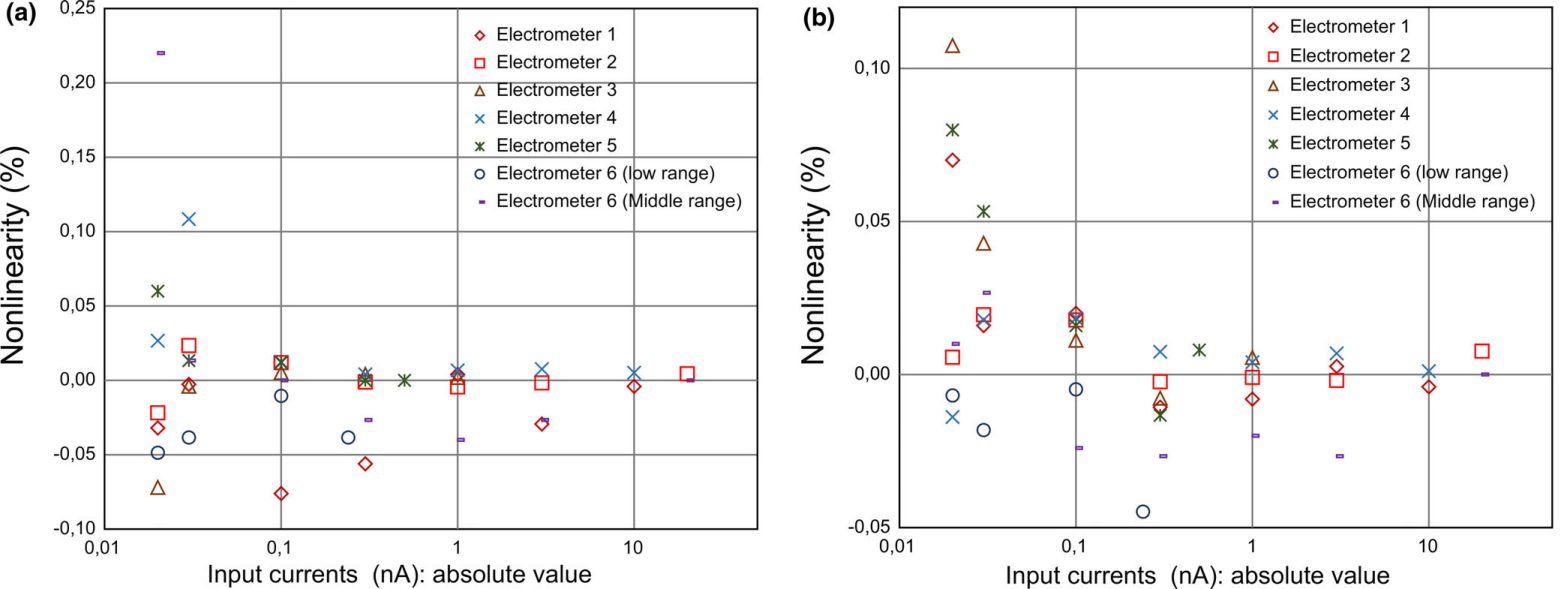
Nonlinearities for the six electrometers when (a) negative and (b) positive currents generated from the DC generator were injected. The y‐axis shows the nonlinearity (%); the x‐axis indicates the injected currents on a logarithmic scale.

The nonlinearities for the six electrometers were usually ±0.05% or less in the range of ±0.1 to ±20 nA (Fig. 3). When constant currents of ±20 and ±30 pA were transferred to the electrometers, the nonlinearities were occasionally ±0.05–0.08% [electrometers 3, 5, and 6 (low range) at –20 pA; electrometers 1 and 5 at 20 pA; and electrometer 5 at 30 pA], and reached 0.1–0.22% in rare cases [electrometer 6 (middle range) at –20 pA; electrometer 4 at –30 pA; and electrometer 3 at 20 pA].

## 4 DISCUSSION

### 4.A Verification of charges from the DC generator

As mentioned in Section 3.A, the measured‐current nonlinearities at 20 pA were usually larger than those at above 20 pA. For example, if a measured current of 20 pA changes only by 10 fA, its nonlinearity is 0.05%. This change could be due to the variation in the generated current caused by the ambient environment (e.g., variations in room temperature and RH due to the use of an air conditioner during the electrometer test) and the electrometer’s zero reading. These variations could have occurred in this work; nevertheless, the measured‐current nonlinearities in all the situations investigated here were well below ±0.09%. These findings suggest that the generated‐current nonlinearities met the performance in the specification.

Differences between the measured charges and set charges were within the uncertainties of the measured charges. These findings suggest that the DC generator generated accurate charges.

### 4.B Electrometer check with the DC generator

The findings described in Section 3.B were not intended to be an indicator of the performance of the six electrometers because the characteristics of the six were different (e.g., date of manufacture, measurement range, design concept). As mentioned in Section 1, this work was a feasibility study of electrometer performance checks in a clinical setting.

Manufacturers evaluate electrometer performance under the test conditions described in IEC 60731:2011. However, clinical environments might differ from manufacturers’ testing conditions. Therefore, the electrometer check results obtained in clinical environments may not agree with the specifications in the catalogs, because of the influence of the ambient environment. When an operator encounters this situation in a clinic, the electrometer should be rechecked to confirm whether the results are reproducible.

#### 4.B.1 Repeatability

As can be seen from Table 5, the repeatability for the six electrometers was within ±0.1%. Because these values were less than ±0.1% despite the variations in the ambient environment, no significant variation would occur in their repeatability.

#### 4.B.2 Nonlinearity

As noted in section 3.B.b, while the nonlinearities from ±0.1 to ±20 nA were usually ±0.05% or less for the six electrometers, those at ±20 and ±30 pA occasionally ranged from ±0.05% to ±0.2% for five of the six electrometers. For example, if an injected current of 20 pA changes by ±40 fA, the nonlinearity at 20 pA is ±0.2%. A current level of 40 fA corresponds approximately to the leakage current of a farmer ion chamber.^7^ This situation may occur in a clinic because of variations in the injected current and the electrometer’s zero reading mentioned above.

The nonlinearity for electrometer 6 was approximately 0.2% at the current of −20 pA. Even when its reading changes from 1.000 to 1.002 nC (electrometer 6 provides four‐digit readings), when an operator measures the current of 20 pA for 50 s, the nonlinearity is 0.1%. If its actual reading was rounded to a large value, the nonlinearity might be less than 0.2%.

In this work, although the nonlinearities at 20 and 30 pA were typically somewhat larger than those for the currents above ±100 pA, our values were within the specifications described in the catalogs for the six electrometers.

## 5 CONCLUSION

At currents of ±20–30 pA, nonlinearity and repeatability may not depend solely on the performance of the electrometer itself; variations in the electrometer’s zero reading and injected current can also play a role. At current levels of 100 pA or more, however, such variations have no significant effect.

## AUTHOR CONTRIBUTIONS

Concepts: Naoki Kinoshita, Hiroshi Oguchi, and Morihito Shimizu. Design: Naoki Kinoshita, Hiroshi Oguchi, and Morihito Shimizu. Definition of intellectual content: Naoki Kinoshita, Hiroshi Oguchi, and Morihito Shimizu. Design: Naoki Kinoshita, Hiroshi Oguchi, and Morihito Shimizu. Literature search: Naoki Kinoshita. Experimental studies: Naoki Kinoshita. Data acquisition: Naoki Kinoshita. Data analysis: Naoki Kinoshita. Statistical analysis: Naoki Kinoshita. Manuscript preparation: Naoki Kinoshita. Manuscript editing: Naoki Kinoshita, Hiroshi Oguchi, Morihito Shimizu, Eiji Kidoya, Hiroki Shioura, and Hirohiko Kimura. Manuscript review: Naoki Kinoshita, Hiroshi Oguchi, Morihito Shimizu, Eiji Kidoya, Hiroki Shioura, and Hirohiko Kimura.

## CONFLICT OF INTEREST

We borrowed the DC generator used in this work from EMF Japan Co., Ltd. We borrowed the electrometers from Chiyoda Technol Corporation, EMF Japan Co., Ltd., and TOYO MEDIC Co., Ltd.

## Data Availability

The data that support the findings of this study are available from the corresponding author upon reasonable request.
